# The Peritoneal Surface Proteome in a Model of Chronic Peritoneal Dialysis Reveals Mechanisms of Membrane Damage and Preservation

**DOI:** 10.3389/fphys.2019.00472

**Published:** 2019-05-14

**Authors:** Michael Boehm, Rebecca Herzog, Florian Klinglmüller, Anton M. Lichtenauer, Anja Wagner, Markus Unterwurzacher, Robert H. J. Beelen, Seth L. Alper, Christoph Aufricht, Klaus Kratochwill

**Affiliations:** ^1^Division of Pediatric Nephrology and Gastroenterology, Department of Pediatrics and Adolescent Medicine, Medical University of Vienna, Vienna, Austria; ^2^Christian Doppler Laboratory for Molecular Stress Research in Peritoneal Dialysis, Department of Pediatrics and Adolescent Medicine, Medical University of Vienna, Vienna, Austria; ^3^Center for Medical Statistics, Informatics, and Intelligent Systems-CeMSIIS, Medical University of Vienna, Vienna, Austria; ^4^Department of Molecular Cell Biology and Immunology, VU University Medical Center, Amsterdam, Netherlands; ^5^Division of Nephrology, Beth Israel Deaconess Medical Center, Boston, MA, United States; ^6^Department of Medicine, Harvard Medical School, Boston, MA, United States

**Keywords:** N(2)-alanyl-L-glutamine, cytoprotective additive, *in vivo* proteomics, mesothelial cells, peritoneal immune response, PD rat model, animal model

## Abstract

Peritoneal dialysis (PD) fluids are cytotoxic to the peritoneum. Recent studies have shown that alanyl-glutamine (AlaGln) modulates the cellular stress response, improves mesothelial cell survival, reduces submesothelial thickening in experimental models of PD, and in clinical studies improves PD effluent cell stress and immune responses. However, the mechanisms of AlaGln-mediated membrane protection are not yet fully understood. Here, we explore those mechanisms through application of a novel proteomics approach in a clinically relevant *in vivo* model in rats. Experimental PD was performed for 5 weeks using conventional single-chamber bag (SCB) or neutral dual-chamber bag (DCB), PD fluid (PDF), with or without AlaGln supplementation, via a surgically implanted catheter. Rats subjected to a single dwell without catheter implantation served as controls. The peritoneal surface proteome was directly harvested by detergent extraction and subjected to proteomic analysis by two-dimensional difference gel electrophoresis (2D-DiGE) with protein identification by mass spectrometry. An integrated bioinformatic approach was applied to identify proteins significantly affected by the treatments despite biological variation and interfering high abundance proteins. From 505 of 744 common spots on 59 gels, 222 unique proteins were identified. Using UniProt database information, proteins were assigned either as high abundance plasma proteins, or as cellular proteins. Statistical analysis employed an adapted workflow from RNA-sequencing, the trimmed mean of *M*-values (TMM) for normalization, and a mixed model for computational identification of significantly differentially abundant proteins. The most prominently enriched pathways after 5 weeks chronic treatment with SCB or DCB, PDFs belonged to clusters reflecting tissue damage and cell differentiation by cytoskeletal reorganization, immune responses, altered metabolism, and oxidative stress and redox homeostasis. Although the AlaGln effect was not as prominent, associated enriched pathways showed mostly regression to control or patterns opposite that of the PDF effect. Our study describes the novel peritoneal surface proteome through combined proteomic and bioinformatic analyses, and assesses changes elicited by chronic experimental PD. The biological processes so identified promise to link molecular mechanisms of membrane damage and protection in the *in vivo* rat model to pathomechanisms and cytoprotective effects observed *in vitro* and in clinical PD.

## Introduction

Peritoneal dialysis (PD) is a life-saving home-based renal replacement therapy which, despite improved preservation of residual renal function, remains underutilized due to its limitations of peritonitis and peritoneal fibrosis, leading to membrane, and technique failure (Davies, 2013). Chronic exposure to PD fluid (PDF) causes injury to the mesothelial cell layer of the peritoneal wall that serves as the dialysis membrane. Included among the contributors to the attendant chronic inflammation are deficient induction of cytoprotective cell stress and repair pathways and local immune dysfunction (Kratochwill et al., 2009, 2011; Bender et al., 2011).

Peritoneal dialysis fluid in single chamber bags (SCB) contain all components of the solution in a single compartment. The pH around 5.2 is a compromise between lower formation of glucose degradation products (GDP) at lower pH and damage to the peritoneum including infusion pain. In dual chamber bags (DCB), in contrast, glucose is separated from buffer components during heat sterilization (Garcia-Lopez et al., 2012). The initial acid pH minimizes formation of GDP, while the combined solution instilled into the patient’s peritoneal cavity is restored to neutral pH. Although DCB PDFs cause reduced *in vitro* damage to cells (Topley et al., 1996; Jorres et al., 1998; Del Peso et al., 2015), these fluids may also be less potent inducers of beneficial cell stress, and repair pathways (Schmitt and Aufricht, 2016). This deficiency can result in chronic inflammation, complement activation and increased vascularity, likely underlying the persistent lack of clinical evidence for DCB PDF superiority (Bartosova et al., 2018; Schaefer et al., 2018). Taken together, data from the last 20 years of PD research support the idea that enhancement of beneficial cell stress and repair mechanisms (while in parallel countering chronic inflammation) holds greater promise for reduction of peritonitis and peritoneal fibrosis than does reduction in PDF toxicity.

Alanyl-glutamine (AlaGln) is a substance that has the potential to accomplish this goal. Our group has demonstrated *in vitro* that AlaGln modulates the cellular stress response and improves survival of mesothelial cells (Kratochwill et al., 2012). A first-in-human clinical trial showed that glutamine deficiency during clinical PD is linked to peritoneal pathomechanisms, such as impaired stress response and host defense (Kratochwill et al., 2016). A pilot trial indicated improved PD effluent cell function with regards to stress and immune responses due to priming of effluent cells by AlaGln and reducing basal chronic inflammation (Herzog et al., 2017). The recently conducted multicenter phase II trial confirmed protective effects of AlaGln at the level of surrogate markers of peritoneal membrane status and immune competence (Vychytil et al., 2018). Promising results in this trial regarding decreased peritoneal protein loss require in depth analysis of the potential membrano-protective mechanism.

Evaluating the molecular mechanism of the effect of AlaGln on peritoneal membrane tissue would require biopsies taken from patients after extended periods of treatment with AlaGln-supplemented PDF (and from matched controls). In the meantime, chronic animal models help improve our understanding of peritoneal transport and of peritoneal immune and inflammatory molecular processes in PD therapy and support the selection process for suitable surrogate markers. Indeed, Ferrantelli et al. (2016) has reported reduced submesothelial thickening and decreased vascularization by AlaGln in animal models of PD. Whereas in these studies the focus was on individual candidate markers of chronic inflammation, proteomic analysis would provide a holistic view of the complex molecular dynamics influencing the peritoneal membrane and an attractive approach to understand the molecular mode-of-action of AlaGln.

A recent proteomics study provided unprecedented insight into the composition of PD effluent and the molecular mode-of-action of AlaGln in a single dwell with conventional SCB PDF (Herzog et al., 2018). Nevertheless, this information is a global heterogeneous mix of the plasma proteome and the secretome of peritoneal cell populations, containing greater numbers of leukocytes than of mesothelial cells.

In contrast to human studies relying mostly on PD effluent, animal models allow sampling of the peritoneal wall surface for proteomic analysis. Here, we apply a proteomic approach in a clinically relevant *in vivo* model to characterize the peritoneal surface proteome and analyze the effect of AlaGln addition to PDF on molecular processes in response to chronic PDF exposure.

## Materials and Methods

Standard chemicals were purchased from Sigma - Aldrich (St Louis, MO, United States) if not specified otherwise.

### *In vivo* PD Model in Rats

**FIGURE 1:**
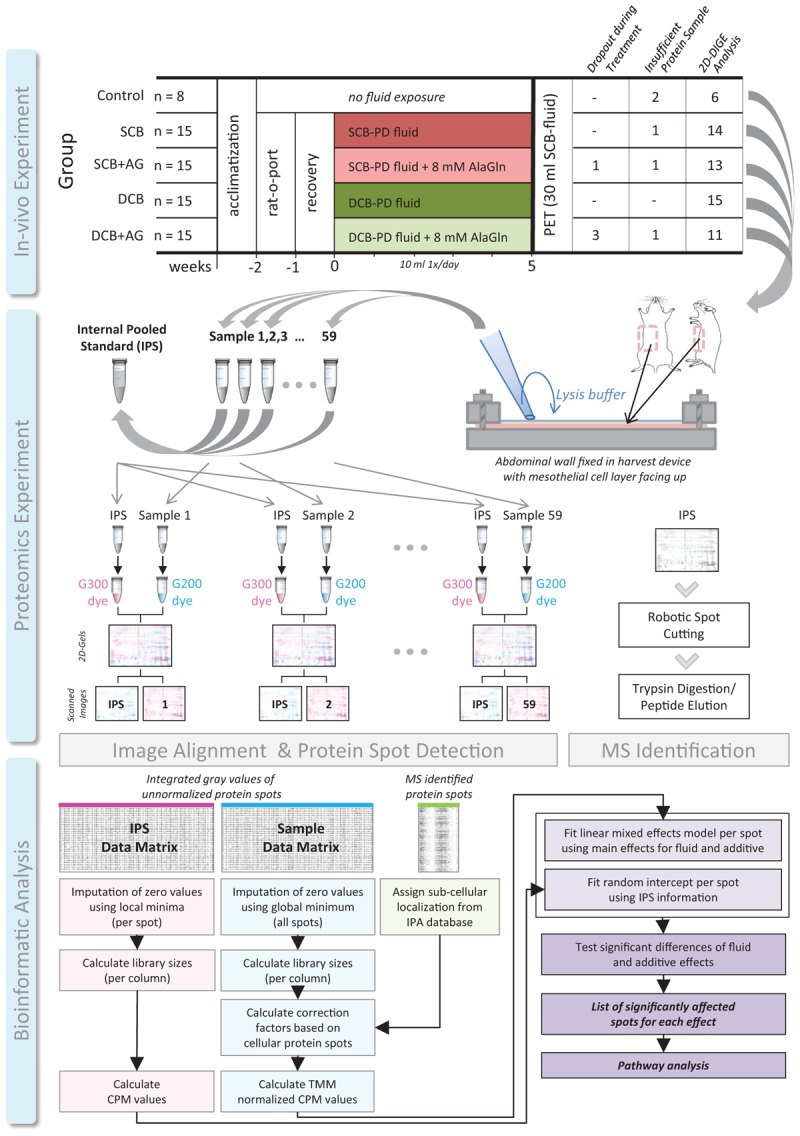
Experimental workflow in three stages. *In vivo* experiment with 5 experimental groups. SCB, single-chamber bag; DCB, dual-chamber bag; AG, alanyl-glutamine; rat-o-port, surgically implanted catheter for PDF instillation; PET, peritoneal equilibration test. Proteomics experiment with steps sample lysis and harvest, preparation of the IPS and labeling of samples, 2D-DiGE, and fluorescent image scanning. IPS, internal pooled standard, G200, and G300 fluorescent dyes used for difference gel electrophoresis. Bioinformatic analysis, following the presented integrated workflow. CPM, counts per million relative signal calculation strategy adapted from RNA-sequencing workflows; TMM, trimmed mean of *M*-values normalization strategy adapted from RNA-sequencing workflows.

Male Wistar rats (13 weeks, 300 g, Harlan; CPB, Horst, Netherlands) were housed under normal conditions, with water and food *ad libitum*. The study was approved by the animal care committee of the Vrije Universiteit of Amsterdam. Rats received, under isoflurane anesthesia and analgesia, a subcutaneously implanted catheter to the peritoneal cavity (“rat-o-port”, Access Technologies, Norfolk Medical, Skokie, IL, United States) for daily installation of PDF. Following 7 days of healing and daily instillation of 2 ml physiological saline (NaCl 0.9%) with 1 U/ml heparin the rats received for 5 weeks daily injections of 10 ml PDF fluid (Figure 1). Rats were exposed to either glucose-based, lactate buffered, acidic pH, SCB PDF (Dianeal PD4 3.86%, Baxter Healthcare, Deerfield, IL, United States; *n* = 15) or the same PDF with added 8 mM AlaGln (Dipeptiven, Fresenius-Kabi, Bad Homburg, Germany) (SCB+AG; *n* = 15) or to glucose-based, bicarbonate/lactate buffered, low GDP, neutral pH, DCB PDF (Physioneal 40 3.86%, Baxter Healthcare, *n* = 15) or the same neutral pH PDF with added 8 mM AlaGln (DCB+AG; *n* = 15). Rats without an implanted catheter (*n* = 8) served as controls and were not subjected to daily PD. Control animals were kept under the same conditions in parallel and were subjected to the PET and harvest on the same day.

Clinical condition of the animals was monitored daily, and weight was recorded weekly. On the last day of the experiment rats received a 90 min peritoneal equilibration test (PET) under anesthesia. 30 ml PDF (Dianeal PD4 3.86%) were instilled, and the abdomen was periodically moved to ensure complete wetting of the peritoneal membrane. After 90 min the PD effluent was drained via a standard venous catheter (Venflon Pro; Becton Dickinson, Franklin Lakes, NJ, United States) placed in the lower right abdomen into the peritoneal cavity, the rats were sacrificed and the abdominal walls were harvested.

### Peritoneal Surface Proteome Isolation

Using a specifically designed harvesting device (Supplementary Figure S1), peritoneal surface proteins were isolated directly from the abdominal walls. The contra-lateral parietal peritoneal wall of the implanted catheter was removed and fixed with the mesothelial cell layer (luminal) side facing up and washed twice (250 mM sucrose, 10 mM Tris–HCl, pH 7.0) to remove residual plasma on the surface. Lysis buffer (500 μl, 250 mM sucrose, 10 mM Tris–HCl, pH 7.0, 0.25% Triton-X 100) was applied to the surface for 2 min, followed by very gentle scraping with a sterile disposable cell scraper to facilitate cell lysis. The resulting protein lysate was collected by tilting the device and pipetting into a collection tube.

For proteomics analyses, protein samples were precipitated (100% acetone, overnight, -20°C), washed with ethanol and resuspended in 200 μl (30 mM Tris, pH 8.5, 7 M urea, 2 M thiourea, 4% 3-[(3-cholamidopropyl) dimethylammonio]-1-propanesulfonate (CHAPS), 1 mM EDTA, 1 tablet of Complete Protease Inhibitor (Roche, Basel; Switzerland), and 1 tablet of phosphatase inhibitor (PhosSTOP, Roche) per 100 mL. Total protein concentration was determined according to the manufacturer’s instructions (2D Quant kit; GE Healthcare, Uppsala, Sweden).

### Two-Dimensional Difference Gel Electrophoresis (2D-DiGE)

An internal pooled standard (IPS) was prepared containing 47 samples in equal parts and was used in all individual gels. Proteins were labeled with fluorescent dyes (Refraction-2D Labeling Kit; NH DyeAGNOSTICS, Halle, Germany) according to the manufacturers’ protocol with minor modifications. In brief, 30 μg protein of all individual samples (*n* = 59) was mixed with 0.2 nmol reconstituted G-Dye200 and the IPS was mixed with 0.2 nmol G-Dye300 (27 μg per gel). Labeling of the IPS was performed in one batch to achieve a uniform standard. Following the labeling (30 min, on ice in the dark) the reaction was stopped (1 μl of stop solution) and incubated for another 10 min on ice in the dark. For every individual gel, the labeled sample and the labeled IPS were mixed together shortly before rehydration.

#### Isoelectric Focusing

First dimensional protein separation with isoelectric focusing was conducted on a Protean IEF Cell (BioRad, Hercules, CA, United States) with 57 μg of total protein (consisting of 30 μg labeled sample and 27 μg labeled IPS) per immobilized pH gradient (IPG) strip [ReadyStrip IPG Strips 24 cm pH 3-10 non-linear (BioRad)]. The rehydration mix containing both sample and IPS was brought to a final volume of 450 μl and a final concentration of 5 M urea, 0.5%w/v CHAPS, 0.5% v/v Pharmalyte (GE Healthcare), and 12 μl/ml of DeStreak reagent (GE Healthcare). The strips were overlayed with mineral oil (Bio-Rad) and actively rehydrated at 50 V for 12 h and afterward focused by increasing the voltage step by step up to 8000 V within 17 h. The procedure was carried out at 20°C using a current limit of 30 μA per strip. Focused strips were stored at -80°C until further use.

#### Horizontal Gel Electrophoresis

The second dimension of protein separation was performed on a HPE-FlatTop Tower (Serva Electrophoresis GmbH, Heidelberg, Germany) following the manufacturer’s manual using precast non fluorescent gels (HPE Large Gel NF 12.5% Kit, Serva). The proteins in the focused IPG strips were reduced and alkylated by consecutive incubation in equilibration buffer (Serva) with 1.8 g/5ml urea mixed with 50 mg/5ml dithiothreitol (DTT) for the first and with 125 mg/5ml iodoacetamide (IAA) for the second 25 min of incubation. The gels were placed onto the cooling plates with equal amounts of cooling contact fluid (Serva) beneath the gel and anode or cathode buffer soaked electrode wicks (Serva) were placed on the gels’ edges (2 mm overlapping). The equilibrated IPG strips were applied on the gels and the electrophoresis was performed in five steps at 15°C according to the manufacturer’s manual. The protocol started with 100 V, 28 mA, 4 W for 30 min, followed by 200 V, 52 mA, 12 W for another 30 min. Then 300 V, 80 mA, and 20 W were applied for 10 min. After this step, the IPG strips were removed and the run continued with 1500 V, 160 mA, and 120 W for 3 h 50 min. In a last step 1500 V, 180 mA, and 160 W were applied for another 50 min. Following electrophoresis, the gels were washed (H_2_O) and stored at 4°C until fluorescence image acquisition.

#### Fluorescence Image Acquisition and Data Analysis

Gel images with fluorescent signals were acquired using a laser scanner (ThypoonTrio, GE Healthcare) at the labeling kit manual’s recommended excitation and emission wavelengths (G-Dye200: ex/em 554/575 nm; G-Dye300: ex/em 648/663 nm). The photomultiplier voltage was adjusted for near saturation of the most abundant spots.

#### 2D Gel Image Analysis

Gel images were analyzed using the Delta2D 4.2 software (Decodon GmbH, Greifswald, Germany) with the algorithm designated for DiGE experiments. IPS images were aligned by pair-wise warping, and spot detection was carried out on a fused image of all gels.

### Protein Spot Identification by Matrix-Assisted Laser Desorption/Ionization (MALDI) Mass Spectrometry (MS)

Preparative gels for protein identification were loaded with 200 μg of IPS protein, of which 25 μg were labeled as described above. Gels were stained with Coomassie Brilliant Blue (CBB). Following electrophoresis protein spots were fixed overnight (10% acetic acid, 40% ethanol) followed by 3 washing steps (5 min, H_2_O). After overnight incubation with CBB staining solution [8% (w/v) ammonium sulfate, 2% (w/v) orthophosphoric acid (85%), 20% (v/v) methanol, and 1% (v/v) CBB stock solution 2.5% (w/v – Coomassie Brilliant Blue G250 dissolved in H_2_O)] the staining was intensified with incubation for 30 min in 20% ammonium sulfate (in H_2_O) and briefly destained with 10% glycerol, 20% methanol in (in H_2_O). All incubation steps were carried out on a horizontal rotary shaker.

Protein spots were automatically excised (EXQuest Spot Cutter; BioRad). Excised gel plugs were washed [100 mM NH_4_HCO_3_, 100 mM NH_4_HCO_3_/ethanol (1:1) and acetonitrile] until destained. After reduction (10 mM DTT in 25 mM NH_4_HCO_3_) for 1 h at 56°C, the samples were alkylated (55 mM IAA in 25 mM NH_4_HCO_3_) for 45 min at room temperature in the dark. Following another wash step (100 mM NH_4_HCO_3_ and acetonitrile), samples were digested with 0.39 μg trypsin in 50 mM NH_4_HCO_3_ overnight at 37°C. The cleaved peptides were eluted from the gel plugs with sonication in acetonitrile/H_2_O/trifluoroacetic acid (TFA) (50:45:5). Eluates were dried by vacuum centrifugation (Concentrator plus, Eppendorf, Hamburg, Germany) and the peptides were redissolved with 0.1% TFA and desalted with C18 material (C18-ZipTip columns, Millipore; Billerica, MA, United States) according to the manual. Briefly, the material was wetted with acetonitrile and equilibrated with 0.1% TFA. Peptides were loaded onto the column, followed by washing with 0.1% TFA and direct elution onto two spots of the MALDI target (Thermo Fisher Scientific, Bremen, Germany) with α-cyano-4-hydroxycinnamic acid (10 mg/ml; CHCA; LaserBio Labs, Sophia-Antipolis Cedex, France) in acetonitrile/0.1% TFA.

Mass spectrometric (MS) analyses were performed on a matrix-assisted laser desorption ionization (MALDI) LTQ Orbitrap XL mass spectrometer (Thermo Fisher Scientific) operated in positive mode. MS spectra were acquired in a mass range from m/z 600–4000 with a resolution setting of 100,000 at m/z 400. Acquisition parameters were: automated spectrum filter off, automated gain control on, crystal positioning system on and 5 scans/step. 10 MS spectra were acquired for each spot. For tandem mass spectrometry (MS/MS) the mass spectrometer was operated in a data-dependent mode. Utilized parameters included precursor ion isolation in the linear ion trap, 3 mass units isolation width, 3 normalized collision energies (CID; 30, 35, and 40%), activation q of 0.25 and activation time of 30 ms. The 15 most prominent ions were sequentially isolated for collision-induced dissociation (CID) fragmentation in the linear ion trap.

The acquired raw MS data files were processed (Mascot Distiller 2.7.1.0; Matrix Science, London, United Kingdom) and searched against the rat SwissProt database (Rat_20170830) using Mascot. Additional search parameters were, enzyme: trypsin/P; allowed missed cleavages: 2; fixed modifications, carbamidomethyl (C); variable modifications, oxidation (M); peptide tolerance, 5 ppm; MS/MS tolerance, 0.8 Da; charge state, 1+, 2+, or 3+. The ions score was set to 20 and standard scoring was chosen. Single peptide identifications, which were not supported by another spot, were checked manually by the following criteria: reviewed/unreviewed, molecular weight and *p*I, length of peptide, score, and annotation of b- and y-ions. One protein was excluded from the results list due to wrong MW, no identified y-ions and unreviewed status.

### Scaling and Normalization Approach for Derivation of Protein Spots Associated With the Effect of PDF Exposure and AlaGln Addition

Spot intensity data for each gel were exported from the Delta 2D software and imported in R as a matrix containing all intensity data for sample channels, as well as internal standard channels.

The image analysis workflow supported in the Delta 2D software ensures a data matrix that is free from missing values. The detection limits of the fluorescence dyes and the laser scanner and rounding errors result in the presence of zero values in the data matrix. This impedes further raw data processing using linear mixed model analysis (LIMMA), because the LIMMA requires log-transformed data. Zero values in the data matrix were therefore replaced with the lowest observed value in the complete matrix (sample channel) or the lowest value observed for the protein spot (IPS channel).

To account for inter-individual variation introduced by plasma proteins, a statistical workflow from RNA sequencing (RNAseq), based on trimmed mean of *M*-values (TMM) (Robinson and Oshlack, 2010) was adapted for 2D-DiGE data. Analogous to varying library size in RNAseq, the plasma-type protein spots disproportionately influence the abundance calculation of cellular-type protein spots, an effect that can be observed in many omics-type data. By scaling the spot data based on library sizes calculated only from cellular-type spots, instead of all spots as in the standard workflow, this effect was removed from the data (Figure 1).

Separate linear mixed effects models were fit to the normalized log-transformed spot data of each protein, taking into account the effect of PDF exposure (fluid effect) with or without AlaGln (additive effect) as opposed to sham treatment using fixed effects, along with a random intercept term representing normalization by the IPS. Fold-changes associated with individual effects were extracted from the linear model. For data visualization, random intercepts were subtracted from TMM-normalized values. Significance values were derived from group comparisons utilizing *t*-tests with the obtained *p*-values as well as BH-corrected *q*-values given in Supplementary Table S2.

### Statistical Analysis and Data Visualization

All statistical analyses and visualizations used R (v3.5.1^1^). Ingenuity Pathway Analysis (IPA 7.0, Qiagen^2^) was used to identify pathways and predicted up/down regulation patterns significantly affected by differentially abundant proteins, calculating a *p*-value for each functional pathway using a one-tailed Fisher exact test. Pathways with *p*-values <0.05, after correction for multiple hypothesis testing with the Benjamini-Hochberg (BH) procedure, were considered significantly enriched. The IPA z-score assesses the match of observed and predicted up/down regulation patterns and serves a predictor for the activation state.

### Data Availability

All 2D-DiGE datasets for this study are included in the manuscript and the Supplementary Files. The raw instrument files of MALDI-MS identifications will be made available by the authors, without undue reservation, to any qualified researcher. The R code that was used to generate the analysis is disclosed as Supplementary Material.

## Results

### Experimental Groups

Sixty-four of 68 rats completed the proposed protocol. Four animals failed to complete the chronic dialysis regiment, and in one animal the PET could not be conducted. Due to leakage of the harvesting device, the samples obtained from a few animals were either insufficient in volume or contaminated and thus not used for 2D-DiGE analysis. Final sample group sizes subjected to proteomic analysis were 6 controls, 14 in the SCB group, 13 in the SCB+AG group, 15 in the DCB group, and 11 in the DCB+AG group (Figure 1; see Supplementary Table S1 for details on dropouts and samples with insufficient volume).

### Protein Separation and Identification Workflow

**FIGURE 2:**
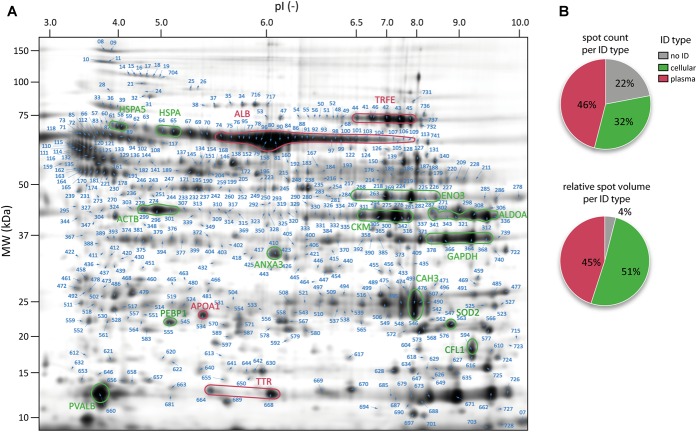
Map of the peritoneal surface proteome. **(A)** Representative 2D gel (Fusion Image) of peritoneal surface proteome of rats. All identified spots are indicated with an arrow and their spot label. Prototypical intense cellular and plasma spots are marked and labeled with the gene name. Cellular proteins are highlighted in green and plasma proteins in red. **(B)** Pie-charts showing the spot counts (upper panel) and relative spot volumes (lower panel) for cellular and plasma proteins.

Analysis of sample homogeneity based on raw spot intensity data revealed experimental variation in the data that exceeded the level typically observed in 2D-DiGE data (e.g., from cell culture samples). Although the overall technical quality of the gels was excellent (Supplementary Figure S2), the harvested samples clearly included materials originating not only from mesothelial cells but also plasma protein contaminants. The gel images exhibit a spot pattern that resembles both cellular-type samples, as evidenced by major spots being cytoskeletal proteins, but also plasma-type samples indicated by prototypical high abundance spots. Nevertheless, spots attributable to albumin show clearly reduced abundance compared to typical gels from serum (Figure 2A).

In contrast to a standard workflow in which only significantly altered protein spots are forwarded to MS identification, in this experiment abundance alterations in cellular proteins would not be detectable without subtraction of plasma protein contaminants. We therefore aimed to identify as many spots as possible of the total protein spot pattern in order to generate a comprehensive map of the peritoneal surface proteome. From the total pattern of 744 protein spots, 625 were above the threshold of intensity and spot quality and were therefore cut and analyzed by MALDI-MS. In 505 of these spots (80%), proteins were successfully identified (Figure 2A), linking to 222 unique protein IDs (Supplementary Table S2).

Information on subcellular localization of all identified proteins was extracted from IPA, allowing protein assignment either to the group of high abundance plasma proteins (plasma type) or to the group of cellular proteins (cellular type) (Supplementary Table S2). The proportion of cellular and plasma proteins is shown in Figure 2B. Computing this proportion, considering spot abundance (integrated gray volume) confirms that these spots can indeed shift the abundance distribution in favor of plasma protein-type spots. The data also show that spots without successful protein identification were on average of very low abundance.

To account for inter-individual variation introduced by plasma proteins, a statistical workflow that has been introduced for RNAseq data, but is equally suitable for matrix from any omics-type experiment, based on TMM (Robinson and Oshlack, 2010) was adapted for 2D-DiGE data. Standard 2DE preprocessing typically subtracts the amount of protein volume measured on the internal standard channel from the corresponding measurement on the sample channel. This assumes that the two amounts are measured on the same scale. This assumption however, does not hold if the proportion of plasma proteins differs between samples but not – as by design – between internal standards (i.e., spot volumes are expressed relative to different amounts of plasma protein). Consequently, adjusting for varying plasma protein content needs to be performed before information from the internal standard can be used to reduce variability arising from systematic differences between gels. Analogous to varying library size in RNAseq, spots on 2D gels that contain plasma-type proteins that are in the state of transcellular migration (or adhere to peritoneal surface denuded from mesothelial cells) and are thus “dissolved” in the tissue, may disproportionately influence the abundance calculation of cellular-type protein spots. By scaling the spot data based on library sizes calculated only from cellular-type spots, this influence was removed from the data during the TMM normalization step. The TMM normalization step (with and without the utilization of the linear model) was compared to a standard 2D-DiGE workflow which was prone to yield more statistically significant spots for the comparisons of the fluid effect and at the same time was less sensitive to the subtle changes of the additive effect (Supplementary Figure S3). We fit a random intercept for each protein using measurements from the sample channel and the internal standard. Essentially this is equivalent to subtracting the internal standard measurement from the sample measurement – as with standard processing – with the added benefit that within a linear model definition we have added flexibility to model additional error components, obtain meaningful estimates of treatment effects via linear contrasts, and get diagnostics about the model fit and error terms by means of variance components estimates.

### Evaluation of Changes in Surface Proteome by Chronic PD

**FIGURE 3:**
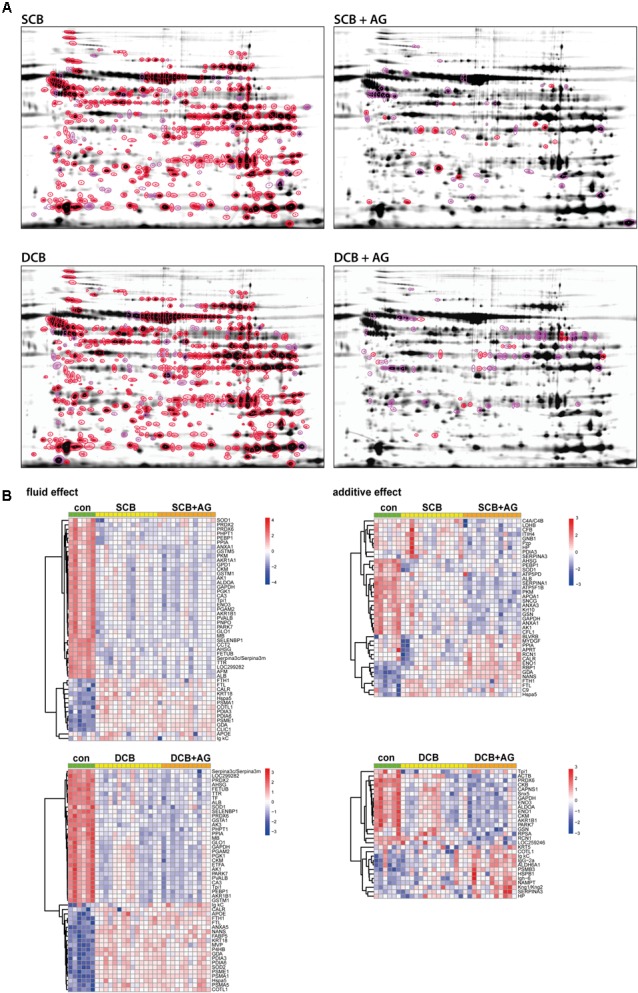
Proteins affected by chronic treatment with PDF and addition of AlaGln **(A)** Representative 2D gel fusion images for each treatment provide an illustration of spot positions, numbers of significant spots, and the technical quality of the gels. They do not represent the dynamic range of intensities of the respective spots. Numeric values for spot abundance are calculated from integration of fluorescent values and taking into account the internal standard for the respective spot. Upper left, SCB; upper right, SCB + AlaGln; lower left, DCB; and lower right, DCB + AlaGln; highlighting significantly altered proteins with *p* < 0.01 in red and with *p* < 0.05 in pink. **(B)** Heatmap showing the top 50 significant (*p* < 0.05) spots with identification for each coefficient (upper left, fluid effect SCB; lower left, fluid effect DCB; upper right, additive effect SCB + AlaGln; and lower right, additive effect DCB + AlaGln). Values are based on TMM-normalized log2 data, with random intercepts subtracted and spots averaged via mean for each molecule (unique protein). Clustering of molecules is based on Pearson-correlation with average agglomeration. Rows were centered and scaled.

Trimmed mean of *M*-values-normalized data were subjected to a linear mixed effects model, considering the effect of PDF (for SCB and DCB groups), and the effect of the AlaGln additive (for SCB+AG and DCB+AG groups) as well as experimental covariates such as the internal standard signal comigrating with every applicable spot on each 2D gel. Using this model, with a threshold of *p* < 0.05, out of all 744 spots 452 protein spots (with 152 unique IDs) were significantly altered by SCB PDF (Figure 3A; numeric values for spot intensities have to be obtained from Supplementary Table S2) and 504 protein spots (with 167 unique IDs) were significantly altered by DCB PDF. 413 of these protein spots with 138 unique IDs were significantly changed by both DCB PDF and SCB PDF. The mixed model analysis identified 63 protein spots with 40 unique IDs as significantly changed in the group treated with AlaGln addition to SCB PDF, and 85 protein spots with 30 unique IDs as significantly changed in the group treated with AlaGln addition to DCB PDF. Hierarchical clustering of significantly altered proteins clearly separated the experimental groups (Figure 3B, top 50 molecules shown). Applying a more stringent threshold of *p* < 0.01 (highlighted in Figure 3 and in Supplementary Table S2) to reduce false positives slightly lowered numbers of proteins significantly changed by PDF effect (by 14% for SCB and by 11% for DCB groups), and to a greater degree for the effect of AlaGln addition (by 72% for SCB and by 78% for DCB groups), indicating an effect magnitude for PDF markedly higher than for AlaGln addition. Consistent with this finding, initially detected changes in numerous proteins remained significant after correction for multiple testing (BH corrected *p* < 0.05: 424 spots for SCB and 494 spots for DCB groups), whereas no spots remained significant for the effect of AlaGln supplementation.

### Pathway Analysis of PD-Related Proteome Changes

To avoid interpretation of false positive candidate molecules, while preserving coverage of relevant biological processes and pathways, a second level of statistical analysis was applied. UniProt accessions of significantly altered spots, together with their respective *p*-, *q*- and fold-change values (see Supplementary Table S2 for details) were forwarded to pathway analysis, condensing the spot information on the level of unique protein coding gene names (molecules). The analysis of significantly enriched canonical pathways was BH-corrected for multiple hypothesis testing, and only proteins passing both abundance and pathway thresholds were interpreted.

**FIGURE 4:**
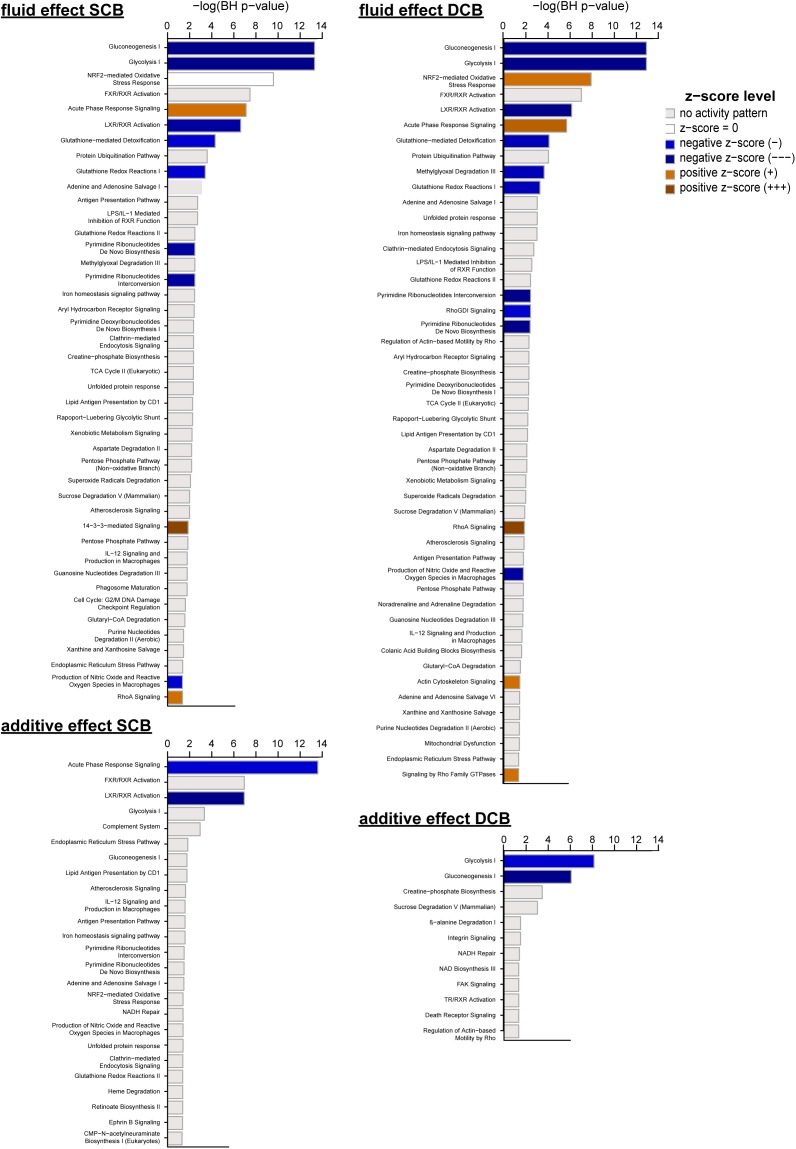
Canonical pathways enriched after chronic treatment with PDF and addition of AlaGln. Significantly enriched canonical pathways from IPA, passing a threshold of *p* < 0.05 after correction for multiple hypothesis testing using the Benjamini-Hochberg (BH) procedure (numeric data are given in Supplementary Table S3). Fluid effect SCB, fluid effect DCB, additive effect SCB, and additive effect DCB denote the effects calculated from the mixed model analysis for protein spot data, which were used for generating the lists of significantly affected molecules for pathway analysis.

Enriched canonical pathways for effects of PDFs and additive addition were extracted and compared for level of enrichment (BH-corrected *p* < 0.05), enrichment ratio and activation z-score (Supplementary Table S3). Figure 4 shows the enriched canonical pathways for the individual effects sorted by enrichment *p*-value (see Supplementary Figure S4 for hierarchical clustering of pathways based on significance of enrichment for all canonical pathways with BH-corrected *p* value < 0.05).

Those canonical pathways for which at least one z-score could be calculated for the four effects (SCB and DCB fluid effects, additive effects for SCB+AG, and DCB+AG), were grouped in functional clusters of typical PD-associated pathomechanisms (metabolism, immune response, cytoskeletal reorganization, and signaling, oxidative stress and redox homeostasis) as well as on molecular features based on shared genes and/or proteins (Table 1).

**Table 1:** Enriched and activated/deactivated canonical pathways for the effects of chronic treatment with PDFs and additive grouped by PD-associated pathomechanisms.

	SCB fluid effect				SCB+AG additive effect				DCB fluid effect				DCB+AG additive effect			
Ingenuity canonical Pathways	*p*-value (BH)	Ratio	z-score	Molecules	*p*-value (BH)	Ratio	z-score	Molecules	*p*-value (BH)	Ratio	z-score	Molecules	*p*-value (BH)	Ratio	z-score	Molecules
**Cytoskeletal reorganization and signaling**																
Actin cytoskeleton signaling	6.92E-02	0.022	2.24	CFL1, EZR, ACTB, GSN, MSN	1.68E-01	0.009		CFL1, GSN	**3.55E-02**	0.026	1.63	CFL1, EZR, ACTB, ARPC3, GSN, MSN	9.33E-02	0.009		ACTB, GSN
RhoGDI signaling	8.51E-02	0.023	-2.00	CFL1, EZR, ACTB, MSN	1.26E-01	0.011		GNB1, CFL1	**3.80E-03**	0.040	-0.38	CFL1, EZR, ACTB, ARPC3, ARHGDIA, ARHGDIB, MSN	2.39E-01	0.006		ACTB
Signaling by Rho family GTPases	7.94E-02	0.020	2.00	CFL1, EZR, ACTB, VIM, MSN	1.79E-01	0.008		GNB1, CFL1	**4.57E-02**	0.024	1.34	CFL1, EZR, ACTB, ARPC3, VIM, MSN	2.60E-01	0.004		ACTB
RhoA signaling	**4.47E-02**	0.032	1.00	CFL1, EZR, ACTB, MSN	2.94E-01	0.008		CFL1	**1.38E-02**	0.040	0.45	CFL1, EZR, ACTB, ARPC3, MSN	2.15E-01	0.008		ACTB
14-3-3-mediated signaling	**1.38E-02**	0.037	0.45	YWHAE, YWHAB, PDIA3, YWHAZ, VIM	2.96E-01	0.007		PDIA3	6.61E-02	0.029	1.00	YWHAE, PDIA3, YWHAZ, VIM				
**Immune response**																
Acute phase response signaling	**7.76E-08**	0.068	1.13	ALB, TTR, FTL, APOA1, SOD2, TF, APCS, AHSG, C9, SERPINA3, SERPINA1, RBP1	**2.51E-14**	0.068	-1.63	C4A/C4B, ALB, HP, FTL, APOA1, ITIH4, AHSG, C9, CFB, SERPINA3, SERPINA1, RBP1	**2.09E-06**	0.063	1.13	ALB, TTR, HPX, FTL, APOA1, SOD2, TF, AHSG, SERPINA3, SERPINA1, RBP1	8.32E-02	0.011		HP, SERPINA3
Production of nitric oxide and reactive oxygen species in macrophages	**4.47E-02**	0.026	-1.34	APOE, ALB, APOA1, APOA4, SERPINA1	**3.98E-02**	0.016		ALB, APOA1, SERPINA1	**1.70E-02**	0.031	-1.63	APOE, ALB, APOA1, APOA4, PPP1R7, SERPINA1				
LXR/RXR activation	**2.40E-07**	0.083	-1.90	APOE, ALB, TTR, APOA1, APOA4, TF, AHSG, C9, SERPINA1, GC	**1.15E-07**	0.058	-1.89	C4A/C4B, ALB, APOA1, ITIH4, AHSG, C9, SERPINA1	**7.24E-07**	0.083	-2.53	APOE, ALB, TTR, HPX, APOA1, APOA4, TF, AHSG, SERPINA1, GC				
**Metabolism**																
Gluconeo genesis I	**5.01E-14**	0.385	-2.53	PGK1, ENO1, PGAM1, ENO3, ENO2, PGAM2, GAPDH, ALDOA, MDH1, MDH2	**1.74E-02**	0.077		ENO1, GAPDH	**1.26E-13**	0.385	-2.53	PGK1, ENO1, PGAM1, ENO3, ENO2, PGAM2, GAPDH, ALDOA, MDH1, MDH2	**8.32E-07**	0.154	-2.00	ENO1, ENO3, GAPDH, ALDOA
Glycolysis I	**5.01E-14**	0.385	-2.53	PGK1, ENO1, PGAM1, ENO3, PKM, ENO2, PGAM2, GAPDH, ALDOA, Tpi1	**4.68E-04**	0.115		ENO1, PKM, GAPDH	**1.26E-13**	0.385	-2.53	PGK1, ENO1, PGAM1, ENO3, PKM, ENO2, PGAM2, GAPDH, ALDOA, Tpi1	**7.08E-09**	0.192	-1.34	ENO1, ENO3, GAPDH, ALDOA, Tpi1
Pyrimidine ribonucleotides *de novo* biosynthesis	**3.39E-03**	0.085	-2.00	AK1, ANXA1, NME2, CMPK1	**3.24E-02**	0.043		AK1, ANXA1	**4.17E-03**	0.085	-2.00	AK1, ANXA1, NME2, CMPK1				
Pyrimidine ribonucleotides interconversion	**3.39E-03**	0.089	-2.00	AK1, ANXA1, NME2, CMPK1	**3.16E-02**	0.044		AK1, ANXA1	**3.80E-03**	0.089	-2.00	AK1, ANXA1, NME2, CMPK1				
**Oxidative stress and redox homeostasis**																
Glutathione-mediated detoxification	**4.90E-05**	0.161	-1.34	GSTM1, GSTM5, GSTM3, GSTA1, GSTP1					**8.13E-05**	0.161	-1.34	GSTM1, GSTM5, GSTM3, GSTA1, GSTP1				
Glutathione redox reactions I	**3.89E-04**	0.167	-1.00	GSTM1, GSTA1, GSTP1, PRDX6					**5.37E-04**	0.167	-1.00	GSTM1, GSTA1, GSTP1, PRDX6	**9.33E-02**	0.042		PRDX6
NRF2-mediated oxidative stress response	**2.63E-10**	0.075	0.00	AKR7A2, GSTM1, AKR1A1, FTL, SOD2, ERP29, GSTM5, PRDX1, ACTB, GSTM3, VCP, GSTA1, SOD1, GSTP1, FTH1	**3.98E-02**	0.015		FTL, SOD1, FTH1	**1.26E-08**	0.070	1.34	AKR7A2, GSTM1, AKR1A1, FTL, SOD2, ERP29, GSTM5, ACTB, GSTM3, VCP, GSTA1, SOD1, GSTP1, FTH1	**2.39E-01**	0.005		ACTB
Methylglyoxal degradation III	**3.39E-03**	0.158		AKR7A2, AKR1A1, AKR1B1					**2.24E-04**	0.211	-1.00	AKR7A2, AKR1A1, AKR1B1, AKR1B10	**8.91E-02**	0.053		AKR1B1

*The table shows canonical pathways for which at least one z-score could be calculated for the four effects (SCB and DCB fluid effects, additive effects for SCB+AG, and DCB+AG – calculated in the mixed model analysis), grouped in functional clusters of typical PD-associated pathways. p-value, The p-value of the overlap was calculated by Fisher’s exact test and corrected by the Benjamini-Hochberg (BH) procedure for multiple testing. The p-values < 0.05 after BH correction are marked in bold font. Ratio, The ratio of pathway enrichment, based on observed vs. expected number of molecules. z-score, The z-score assesses the match of observed and predicted up/down regulation patterns and serves a predictor for the pathway activation state, with positive values indicating activation, and negative values indicating deactivation. Molecules, The molecules observed to be significantly affected by each of the effects, as calculated in the mixed model and that could be assigned to an individual canonical pathway.*

Comparison between SCB and DCB arms of the experiment showed largely overlapping processes and activation states. Concordance was most pronounced for metabolism and immune response pathway clusters, with almost identical activation *z*-scores for involved molecules. Differences in activation pattern (*z*-scores) and involved molecules were more pronounced for the clusters “cytoskeletal reorganization and signaling” and “oxidative stress and redox homeostasis”. Activated “14-3-3-mediated signaling” was found only for the SCB PDF effect, whereas “RhoGDI signaling”, “Signaling by Rho Family GTPases” and “Actin Cytoskeleton Signaling” were activated only for the DCB PDF effect.

The effects for AlaGln additive to both PDFs were not as strong as the fluid effects, indicated by a lower number of significant spots, enriched pathways and calculated *z*-scores. Interestingly, none of the pathways in the cluster “cytoskeletal reorganization and signaling” that were enriched in SCB and/or DCB PDF effects retained significant enrichment for the additive effect. Only the “NRF2-mediated oxidative stress response” pathway reached significance in the cluster “oxidative stress and redox homeostasis”.

The pathways in the “immune response” cluster that remained significantly changed for the AlaGln additive effect included “LXR/RXR activation” and “acute phase response signaling”, with the latter being inhibited by AlaGln.

**FIGURE 5:**
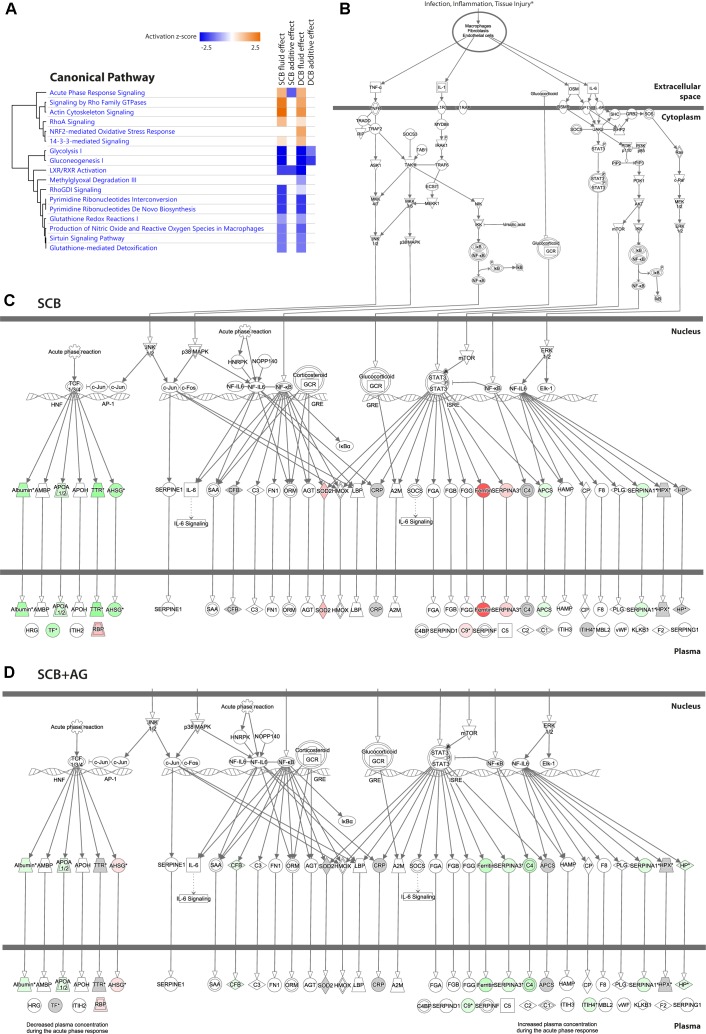
Effect of chronic treatment with PDF and addition of AlaGln on individual pathways, incl. example of “acute phase response signaling”. **(A)** Activation heatmap of enriched canonical pathways from IPA for the four effects calculated from the mixed model analysis. **(B)** Common trunk of the canonical pathway “acute phase signaling”, presented as an example of a significantly enriched pathway in the comparison of both the SCB PDF and additive effects. **(C)** Regulation pattern for the SCB PDF effect. **(D)** Regulation pattern for the SCB+AG effect. Red protein symbols denote upregulated proteins, green protein symbols denote downregulated proteins. Gray protein symbols denote proteins that were identified but not found significantly differentially regulated. Gray protein symbols denote proteins that were not identified in the proteomics experiment.

Figure 5A shows an activation heatmap of enriched pathways. The canonical pathway “acute phase signaling” is presented as an example of a significantly enriched pathway in the comparison of both the SCB fluid and additive effects. Downstream of its common trunk (Figure 5B) the SCB PDF effect (Figure 5C) and SCB+AG effect (Figure 5D) show distinct activation patterns. However, in the presence of AlaGln that pathway was not activated, as indicated by counter-regulation of the same proteins.

## Discussion

We report here the first proteomic analysis of peritoneal surface lysates in a chronic rat model of PD. Proteomics is a particularly attractive approach to understand complex molecular dynamics influencing the peritoneal membrane and to obtain a systems biology view of the molecular mechanisms of membrane damage and preservation during standard PD and following cytoprotective interventions.

Peritoneal dialysis effluent has been previously exploited for indirect investigation of the peritoneal membrane and its secreted proteins. Recent, extensive characterization of PD effluents by a depletion and enrichment proteomics approach (Herzog et al., 2018) yielded signals dominated by extracellular proteins or detached and necrotic mesothelial cells, and complicated by non-resident peritoneal cell populations. Investigating a clean population of resident mesothelial cells in the clinical setting would require peritoneal biopsies in combination with single-cell proteomics techniques currently unavailable. Therefore, initial studies of *in vivo* models must be performed on more complex preparations of peritoneal membrane tissue, such as the optimized lysates prepared with a specifically designed harvesting device for the current study. This novel type of sample likely represents the currently best available peritoneal surface material, comparable only to patient biopsies not easily obtained in the context of experimental interventions beyond the requirements of clinical care (Schaefer et al., 2016).

Rats and mice do certainly not perfectly represent human PD patients. Once daily infusion in the *in vivo* model is contrasted by multiple dwells in the clinical situation. Although uremic rodent models are already available, the use of these models implicitly complicates the experiment by adding technical and biological variation and requiring additional control groups. Nevertheless, rodents exposed to PDF are accepted models to reflect chronic membrane damage and preservation during PD. Recently, glutamine-containing PDF in chronic rodent models showed improved cytoresistance of mesothelial cells in the acute setting, and reduced peritoneal fibrosis and attenuation of IL-17 dependent pathways (Bender et al., 2010; Ferrantelli et al., 2016). The results obtained from rats presented here were generated in an independent replicate experiment to that of Ferrantelli et al. (2016) carried out in the same facility and following identical protocols. Our chronic *in vivo* model of PD led to reproducible submesothelial thickening of the peritoneum (data not shown).

Our proteomics analysis of peritoneal surface lysates has generated the first comprehensive map of the *in vivo* peritoneal surface proteome. We argue that this minimally studied proteome characterizes a functional anatomical entity, analogous to the dermis of the skin or the kidney cortex. As 2D-DiGE analysis preferentially detects high abundance proteins, the ∼500 spots identified in this novel biological sample represent the major protein components of the peritoneal surface. Our relative abundance calculation suggested that identified gel spots represented approximately 95% of sample protein content. Gel images from peritoneal surface isolates resembled a cellular-like pattern, with the typical albumin spot region of significantly lower intensity than seen in typical serum-like patterns. Nevertheless, evidence of plasma proteins persisted in the peritoneal surface proteome data. However, the high reproducibility and technical quality of the internal standard gels suggested that the 2D gel electrophoresis (2D-DiGE) procedure itself was not the source of the variability.

We conclude that the peritoneal surface proteome reported here is not a pure mesothelial cell proteome. Potential explanations for this observation include insufficient washing of the tissue samples, adhesion of plasma proteins to the surface or even below the surface. In case of insufficient washing, the surface lysate sample would be contaminated by residual plasma/fluid. PDE resembles diluted plasma and exhibits a stable composition of major (high abundance) components (Herzog et al., 2018). However, some serum proteins were significantly altered in our experimental groups, indicating systematic effects. Serum proteins tend to bind to cell surfaces and receptor molecules as scaffolding proteins, individually and in complexes, as extensively investigated in the setting of uremia (Duranton et al., 2012), such that gentle washing fails to remove them completely. Sub-surface plasma proteins may represent proteins retained in the inter-cellular space or transported via transcellular transport in the top layer of the tissue, a poorly understood process previously described in PD (Balafa et al., 2011). Plasma protein association in surface tissue might also reflect PDF-induced increase in peritoneal tissue vascularization closer to the peritoneal surface (Williams et al., 2002; Schaefer et al., 2018). Taken together, we can systematically rule out that the observed plasma proteins are mere contamination but can reflect specific pathophysiologic conditions. Any pre-analytic manipulation of the material by depleting plasma proteins might therefore introduce additional unwanted artifacts masking potentially relevant changes of the peritoneal surface proteome.

For this challenge, we devised a method to estimate the proportion of plasma protein using external information about protein and express spot volume in terms of its proportion of the overall protein volume that is not of plasma origin. The TMM procedure is well established to normalize library sizes between samples of RNAseq experiments (Abbas-Aghababazadeh et al., 2018). This is conceptually similar to the processing step that normalizes overall protein amount between gels (Chawade et al., 2014). For our experiment, we have modified this procedure to normalize the overall abundance of non-plasma proteins between samples. Using this statistical workflow that compensated for experimental covariates by normalization and scaling of gel data, along with application of a linear mixed model, we were able to discriminate the effects of chronic PDF exposure and of those of AlaGln addition compared to a single control PET dwell with PDF.

Single-chamber bag and dual-chamber bag peritoneal dialysis fluids produced similar changes in regulation of protein abundance compared to control. Pathway analyses based on proteins differentially expressed after treatment with different fluids are consistent with previously described deleterious effects of PDF. The most prominently enriched processes and pathways after 5 weeks of chronic treatment with SCB or DCB PDFs were attributable to the clusters “cytoskeletal reorganization processes” (reflecting tissue damage and cell differentiation), “immune response”, “altered metabolism” (including but not limited to glucose), and “oxidative stress and redox homeostasis”, suggesting that the chronic rat model represents a good surrogate for chronic clinical PD. Although proteomic modification by the AlaGln effect was not as prominent as that produced by the PDF effect (as indicated by fewer significantly altered protein spots), the AlaGln-associated enriched processes were more closely related to repair processes. Importantly, the protein expression associated with several of the enriched processes changed or even reversed in the AlaGln-treated group, suggesting inactivation by AlaGln of otherwise activated pathways.

Single-chamber bag and dual-chamber bag peritoneal dialysis fluids revealed a similar but non-identical pattern in the cluster of cytoskeletal reorganization pathways, suggesting the cluster’s coverage not only of tissue damage from bioincompatible fluid toxicity, but also actin cytoskeleton signaling related to tissue transformation and cellular *trans*-differentiation. Interestingly, changes in several small GTPase pathways (“Signaling by Rho Family GTPases”, “RhoGDI and RhoA Signaling”) correlated more strongly with DCB than with SCB PDF. Only RhoGDI and RhoA were significant with SCB PDF. RhoGDI signaling was inhibited, whereas other GTPase pathways were activated, despite similar protein patterns, suggesting the observed signaling changes likely represent the effector level, with different upstream regulators eliciting a common phenotype. Also interesting was that 14-3-3-mediated signaling was altered only by SCB PDF. 14-3-3-mediated signaling pathways include several upstream regulators of GSK-3β, activated in immortalized mesothelial cells by the acidic PDFs, Dianeal and Extraneal, but not by the neutralized PDFs, Physioneal and Balance (Rusai et al., 2013). These findings might inform future mechanistic experiments upstream of GSK-3β. The cytoskeletal reorganization pathways were not significantly enriched as a function of AlaGln addition, perhaps reflecting normalization of these processes by AlaGln, or inadequate sensitivity of the proteomic technologies used here. In the case of this “cytoskeletal reorganization” cluster, performance of the harvest PET with the same fluid used for chronic treatment might have been a better choice.

In the cluster of immune response pathways, acute phase response signaling was the central pathway activated by both SCB and DCB PDFs. The pattern of regulation shows upregulation acute phase response activators and downregulation of acute phase response inhibitors, with one prominent exception of retinol binding protein 1 (RBP1, the intra-cellular counterpart of RBP4) which has been identified in almost all studies in PDE, including our own (Herzog et al., 2018). Among activated proteins are components of the complement cascade (also significantly enriched but without calculated z-score). The similarity of SCB and DCB PDFs in acute phase pathway activation patterns is consistent with studies in human biopsies from children on PD with biocompatible fluids (Schaefer et al., 2018). However, the acute response upregulation present at time of harvest might not be maintained at longer equilibration times, since PET duration of the animal experiment was shorter than the typical human dwell time. Indeed, levels of positive acute phase response proteins increase as early as 4–5 h following a single inflammatory stimulus. While this reasoning applies to the control animals, the upregulation observed in the animals undergoing 5 weeks of PD must be considered a chronic effect.

The effect of AlaGln addition is the inverse of the effects of SCB and DCB PDF, leading to inactivation of the acute phase response signaling pathway. In this pathway STAT3 appears to be a signaling hub that is influenced by PDF and AlaGln, perhaps reflecting the differential O-GlcNAcylation (of e.g., STAT proteins) associated with PDF (Herzog et al., 2014). The affected parts of the acute phase response signaling pathway include several complement proteins, and indeed the same effect can be observed for this pathway. Complement system activation was recently reported to be activated by DCB PDFs in correlation with vasculopathy, as shown by transcriptomic and proteomic analysis of pediatric peritoneal biopsies (Bartosova et al., 2018). Down-regulation of the complement system supports the clinical finding that AlaGln addition improves biomarkers of peritoneal health even when combined with biocompatible fluids (Vychytil et al., 2018).

In the cluster of “metabolism” pathways, we found deactivation of glycolysis-associated pathways due to significant downregulation of several glycolytic enzymes in response to SCB and DCB PDF. This is particularly interesting, as SCB and DCB PDF are compared to control and therefore the observed downregulation is between a one-off PET with SCB PDF and chronic treatment for 5 weeks. The downregulation could reflect regulatory adaptation to the chronic high glucose environment and is consistent with earlier *in vitro* studies in which glycolytic enzymes were upregulated following a single exposure to glucose-based PDF, but downregulated compared to glucose exposure alone (Lechner et al., 2010). Future studies should determine if this downregulation is relative to the normal peritoneum (without PET) or represents regression to control levels. The similar SCB and DCB PDF results may reflect their identical glucose concentrations, although an influence of GDP content might have been anticipated. AlaGln addition to DCB PDF down-regulated key enzymes (ENO1, ENO2, GAPDH, and ALDOA) more broadly than when added to SCB PDF (ENO1 and GAPDH) only. This downregulation could indicate adaptation of glycolytic flux with added glutamine, however, the observation of differential effect in DCB PDF needs to be investigated in further studies.

The regulated pathways of the redox cluster include those associated with glutathione turnover, as well as oxidative stress responses linked to the inflammasome and GDP degradation. In particular, the NRF2-mediated oxidative stress response pathway was significantly enriched under both SCB and DCB PDF conditions, but appeared activated only by DCB PDF, based on the differential abundance pattern. The NRF2 pathway was further enriched for the AlaGln effect under SCB conditions, whereas AlaGln was without significant effect on the NRF2 pathway in the presence of DCB PDF. The reason for this difference is unclear, but suggests worthwhile investigative paths addressing the influences of buffer and GDPs. NRF2 is required for L-1β secretion and NLRP3 inflammasome activation (Jhang and Yen, 2017), and the NLRP3 inflammasome can contribute to peritonitis in PD patients (Hautem et al., 2017). This IL-1R-dependent effect was blocked by the IL-1R antagonist, anakinra, which latter also limited damage during PDF-induced sterile inflammation (Kratochwill et al., 2011). The neutral pH DCB solution contains significantly lower amounts of toxic GDP. The methylglyoxal degradation pathway was significantly enriched under both SCB and DCB PDF conditions. However, only after chronic treatment with DCB was this pathway’s activity reduced following a PET with GDP-rich SCB PDF.

Alanyl-glutamine has been shown to mediate beneficial effects when added to PDFs in cell culture and clinical studies (Kratochwill et al., 2012, 2016; Herzog et al., 2017). The first long-term data of AlaGln addition were recently obtained in a multicenter phase II trial (Vychytil et al., 2018), showing improved immune competence as assayed by *ex vivo* stimulated cytokine release of peritoneal leukocytes, and peritoneal membrane protection as indicated by increased CA-125 levels. The fact that CA-125 is the only established biomarker for membrane status in PD reflects our inadequate understanding of membrane damage and protection (Aufricht et al., 2017). *In vitro* mechanistic work suggests a role of protein post-translational modification with O-GlcNAc in mesothelial cell cultures exposed to AlaGln-supplemented PDF (Herzog et al., 2014), but AlaGln effects on the peritoneal membrane have not yet been studied directly. Future studies with human biopsy material will be needed to validate the relevance of our findings in the clinical setting of PD.

The presence of serum proteins among the pathways altered by AlaGln was not expected, since the initial goal of this harvesting procedure was to obtain a pure mesothelial cell sample. However, the systematic effects of PDF and of AlaGln on the lysate levels of these proteins suggests the possibility that their presence may indicate more than plasma contamination or insufficient pre-lysis washing, perhaps even enabling study of paracellular protein transport.

The PET before harvest of the peritoneal surface lysate used SCB PDF in all arms of the chronic experiment, including control, DCB, and SCB. The options for the experiment were performing the PET with same fluid as the chronic treatment or with the same fluid in all groups. In this study, we decided for the latter. The advantages are the absence of matrix effects and improved the ability to compare isolated effects of the chronic treatment phase. The approach may have blunted differences between the solutions. The differences demonstrated may therefore be even more pronounced without PDF fluid type switch. This fluid type switch during the PET may also have induced short term changes. The 90 min PET is probably too short for changes in the protein profile of the surface tissue, but some effluent proteins such as cytokines may be sensitive to this switch, and due to rapid mesothelial secretion could influence the between-group differences generated during the chronic phase. The exposure of control animals to glucose-based PDF for the first time in our protocol may have increased induction of the stress response (Kratochwill et al., 2016). This condition resembles a first exposure of cultured cells to PDF and might explain why *in vitro* biocompatibility experiments often fail to replicate clinical outcomes. In one of the first proteomics studies we indeed showed that mesothelial cells exhibit a more adequate stress response to first SCB PDF exposure than to a subsequent repeat exposure (Kratochwill et al., 2009). Comparison of chronic treatment in rats to control may therefore be analogous to comparison of the stress response in patients on chronic PD compared to their initial exposure to PDF (when the stress response is physiologically active).

Further limitations of the experimental setting include the absence of a catheter in the control group. Our model only compares the effect of chronic PD (incl. a catheter) to the state of the healthy peritoneum. Including a catheter control would have enabled separating the effects of the fluid from the effects of the catheter alone but would also have doubled the amount of comparisons. Finally, the animals were not uremic, as nephrectomy would have increased the experimental complexity and variability and would have required additional controls. Investigating the effect of uremia on the observed peritoneal surface processes may be enabled by this study in combination with clinical material, such as biopsies (Bartosova et al., 2018) or PD effluent (Herzog et al., 2018) from patients with varying levels of uremia.

This study is the first description of the peritoneal surface proteome during chronic PD in a well-established rat model of PD. Combined proteomic and bioinformatic investigation of the effects of different PDFs and of AlaGln supplementation in a chronic rat model proved feasible, despite considerable biological variation in this *in vivo* model. The proteomic method allowed extensive characterization of the peritoneal surface composition and biological processes occurring during chronic PD. By normalizing signals from cellular proteins separately from plasma proteins, assessment of PDF exposure-induced changes in the stress proteome of resident peritoneal mesothelial cells was feasible. Chronic PDF exposure in the *in vivo* rat model activated pathways associated with tissue damage and cell differentiation, immune responses, altered metabolism, and oxidative stress. AlaGln addition attenuated deleterious processes associated with membrane damage, and activated processes linked to membrane protection, consistent with recent *in vitro* and clinical findings.

## Ethics Statement

This study was carried out in accordance with the recommendations of the European Union Guideline on Animal Experiments. The protocol was approved by the animal care committee of the Vrije Universiteit of Amsterdam.

## Author Contributions

MB contributed to the *in vivo* experiment, sample preparation, data analysis and interpretation, and manuscript preparation. RH contributed to the sample preparation, data analysis and interpretation, and manuscript preparation. FK contributed to the statistical data analysis. AL and AW contributed to the 2D-DiGE and MS analysis. MU contributed to the data analysis. RB contributed to the supervision of *in vivo* experiments and critical reading of the manuscript. SA contributed to the data interpretation and critical reading of the manuscript. CA contributed to the study concept, data interpretation, and critical reading of the manuscript. KK contributed to the study concept, data analysis and interpretation, and manuscript preparation.

## Conflict of Interest Statement

CA is co-founder of Zytoprotec GmbH, a spin-off of the Medical University Vienna that holds the patent “Carbohydrate-based peritoneal dialysis fluid comprising glutamine residue” (International Publication Number: WO 2008/106702 A1). RH, AL, AW, MU, and KK are former employees of Zytoprotec GmbH. The remaining authors declare that the research was conducted in the absence of any commercial or financial relationships that could be construed as a potential conflict of interest. The handling Editor declared a past collaboration with the authors.

## Footnotes

1 http://www.r-project.org/

2 http://www.ingenuity.com
