# Supplementary material for: The Peritoneal Surface Proteome in a Model of Chronic Peritoneal Dialysis Reveals Mechanisms of Membrane Damage and Preservation
Source: Front Physiol. 2019 May 14;10:472. doi: 10.3389/fphys.2019.00472 (PMC6530346; doi:10.3389/fphys.2019.00472)
Supplement: Supplementary file 4 [file Data_Sheet_1.PDF]

Supplemental Material

The Peritoneal Surface Proteome in a Model of Chronic Peritoneal Dialysis Reveals Mechanisms of Membrane Damage and Preservation

Michael Boehm[1], Rebecca Herzog[1,2], Florian Klingelmüller[3], Anton M. Lichtenauer[1], Anja Wagner[1,2], Markus Unterwurzacher[1,2], Robert H. J. Beelen[4], Seth L. Alper[5,6], Christoph Aufricht[1], Klaus Kratochwill[1,2]

[1] Division of Pediatric Nephrology and Gastroenterology, Department of Pediatrics and Adolescent Medicine, Medical University of Vienna, Vienna, Austria

[2] Christian Doppler Laboratory for Molecular Stress Research in Peritoneal Dialysis, Department of Pediatrics and Adolescent Medicine, Medical University of Vienna, Vienna, Austria

[3] Center for Medical Statistics, Informatics, and Intelligent Systems-CeMSIIS, Medical University of Vienna, Vienna, Austria

[4] Department of Molecular Cell Biology and Immunology, VU University Medical Center, Amsterdam, The Netherlands

[5] Division of Nephrology, Beth Israel Deaconess Medical Center, Boston, MA, USA

[6] Department of Medicine, Harvard Medical School, Boston, MA, USA

Running title: The Peritoneal Surface Proteome

***Corresponding author:**

Priv.Doz. DI Dr. Klaus Kratochwill

Division of Pediatric Nephrology and Gastroenterology, Department of Pediatrics and Adolescent Medicine, Medical University of Vienna

Währinger Gürtel 18-20, AT-1090 Vienna

Phone: +43/1/40400-73747

Fax: +43/1/40400-73598

E-mail: klaus.kratochwill@meduniwien.ac.at

<u>Supplemental Figure S1</u>: The peritoneal surface harvesting device.

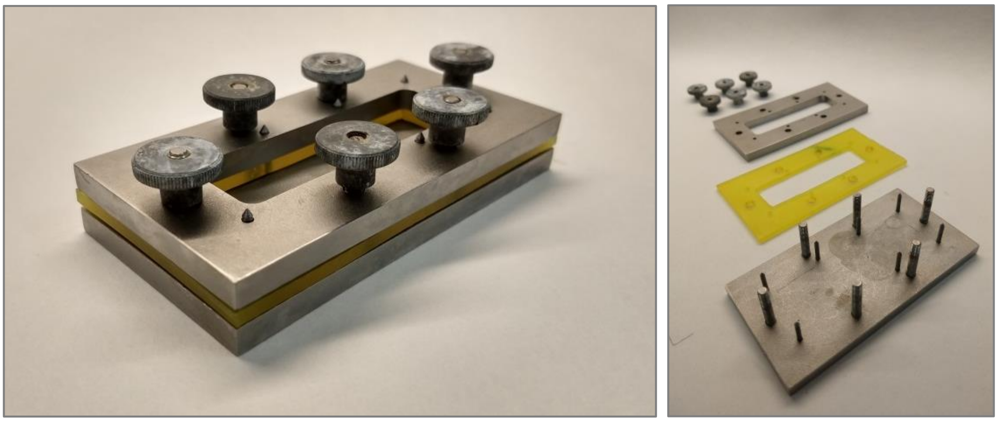

Supplemental Figure S2: all acquired gel images Gel images of peritoneal surface proteome of all rat samples (including samples and IPS) **-** see separate high-resolution file

Supplemental Figure S3: see next page

Supplemental Figure S4: p-values heatmap of enriched canonical pathways from IPA for the four effects calculated from the mixed model analysis - see separate high-resolution file

Supplemental Figure S3: Comparison of TMM normalization and standard 2D-DiGE normalization.

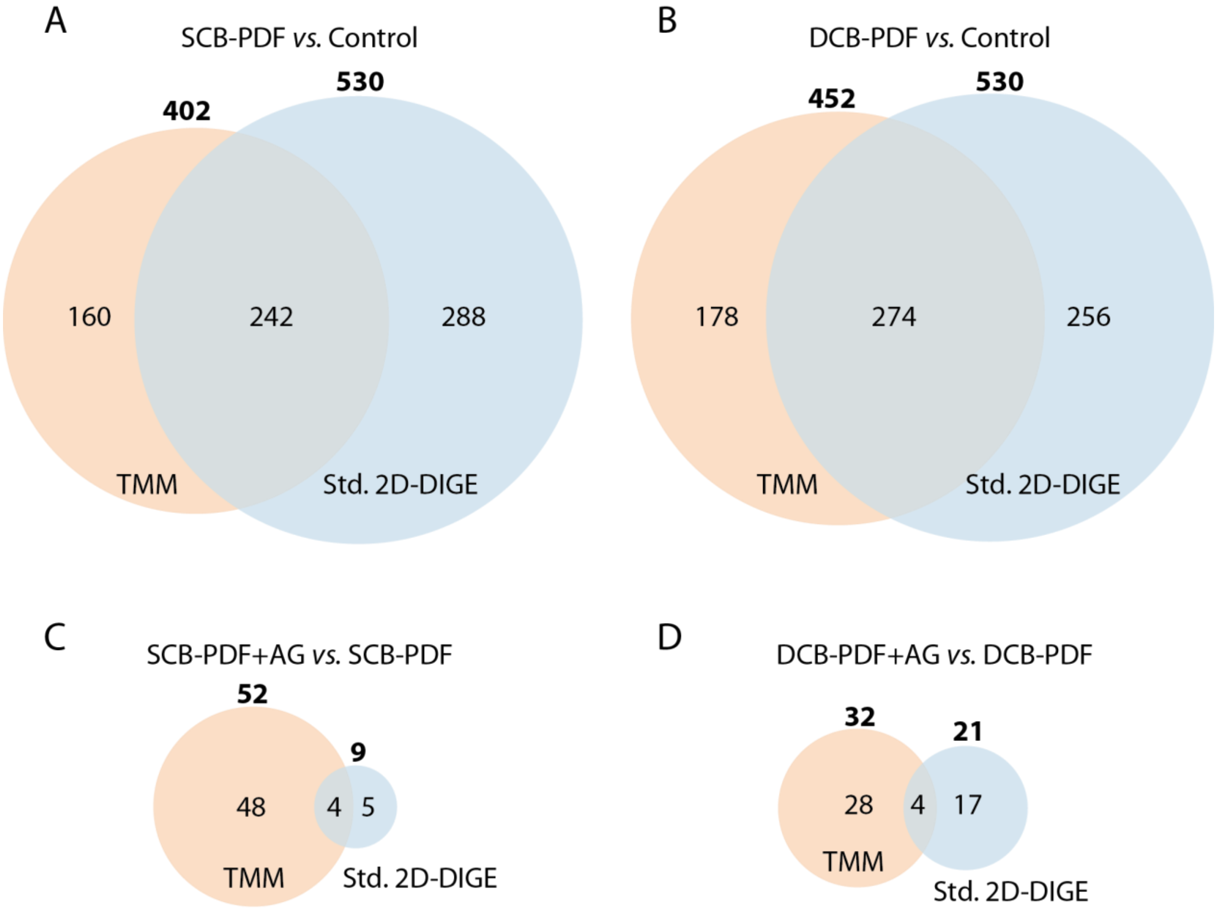

Legend: A naïve standard 2D-DIGE analysis workflow (blue circles) would yield even more statistically significant spots for the comparison of chronic PD *vs.* control (panels A and B) than the TMM normalization (orange circles). For the additive effect (panels C and D), in contrast, simple group-wise t-testing would have identified numbers that are not suitable for biological interpretation via pathway analysis due to lacking sensitivity. Also, due to the shift in relative spot abundance, introduced by high abundance plasma proteins, the numerical values for the fold change of cellular proteins would not be correct. Applying the TMM normalization increases the number of significant spots for the additive effect (480% increase for SCB and 52% increase for DCB) but not for the fluid effect (24% decrease for SCB and 15% decrease for DCB), demonstrating that our chosen approach is more sensitive.
