# Supplementary figures and images for: The Peritoneal Surface Proteome in a Model of Chronic Peritoneal Dialysis Reveals Mechanisms of Membrane Damage and Preservation
Source: Front Physiol. 2019 May 14;10:472. doi: 10.3389/fphys.2019.00472 (PMC6530346; doi:10.3389/fphys.2019.00472)

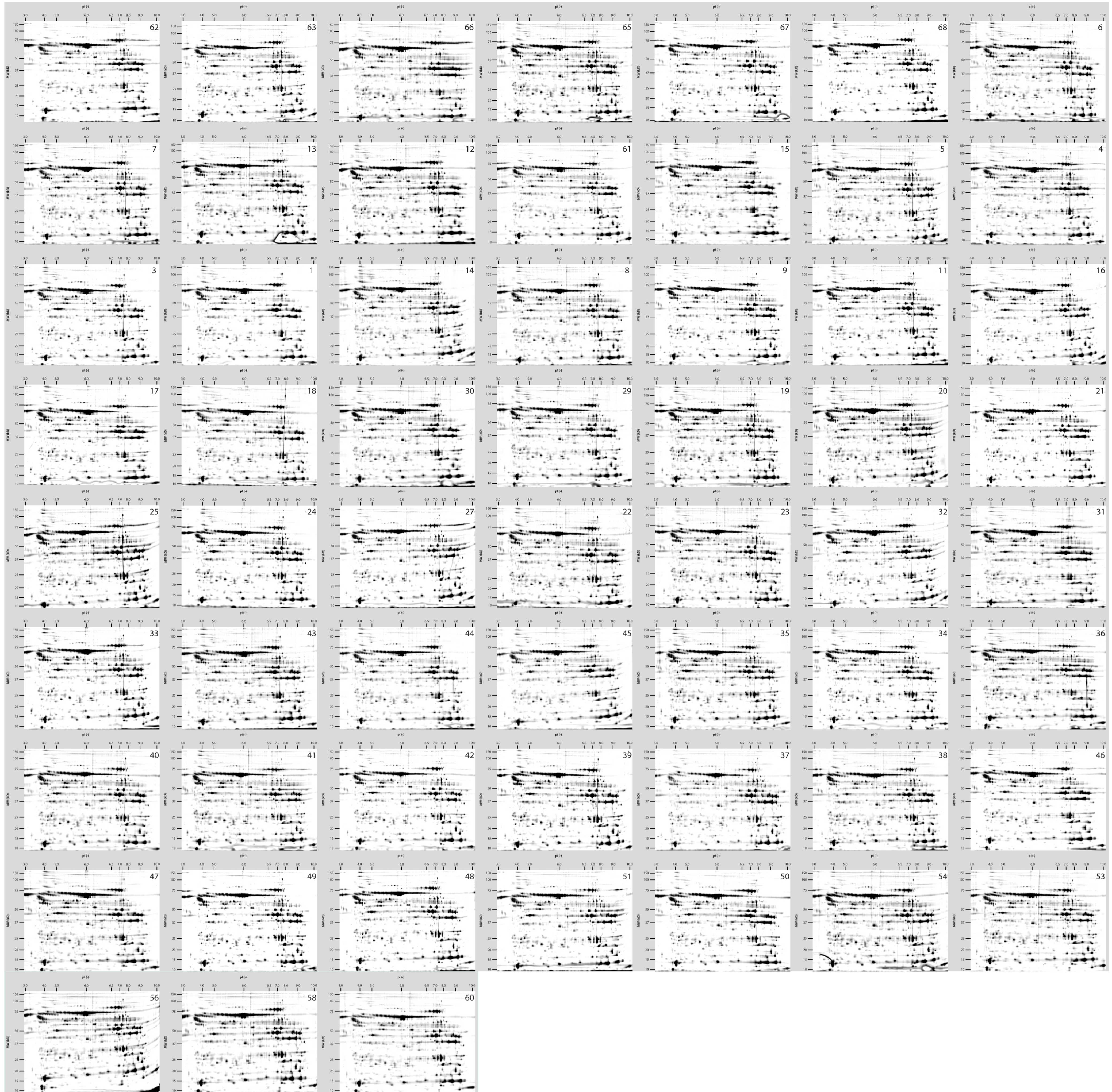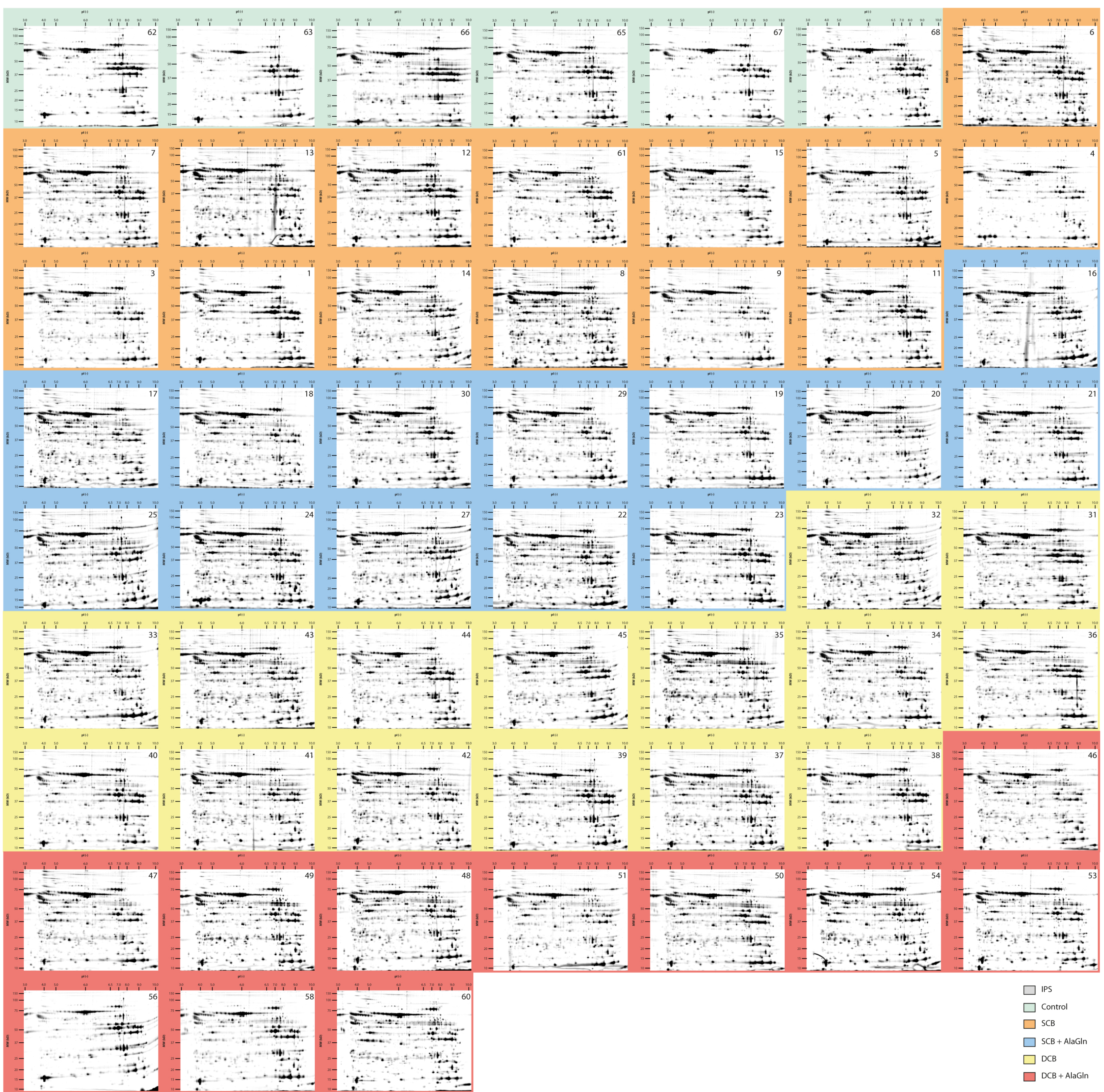

Legend:

- IPs
- Control
- SCB
- SCB + AlaGln
- DCB
- DCB + AlaGln

Supplement: Supplementary file 5 [file Data_Sheet_2.PDF]

Supplemental Figure S3:

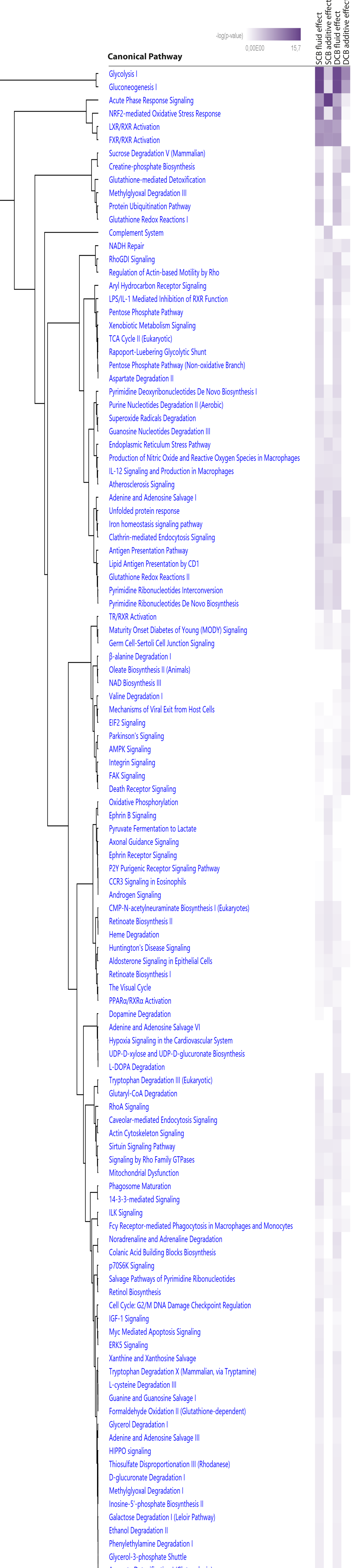

Supplement: Supplementary file 6 [file Data_Sheet_3.PDF]
